# Machine Learning Models in Drilling of Different Types of Glass-Fiber-Reinforced Polymer Composites

**DOI:** 10.3390/polym15234609

**Published:** 2023-12-03

**Authors:** Katarzyna Biruk-Urban, Paul Bere, Jerzy Józwik

**Affiliations:** 1 Department of Production Engineering, Mechanical Engineering Faculty, Lublin University of Technology, 20-618 Lublin, Poland; j.jozwik@pollub.pl; 2 Department of Manufacturing Engineering, Faculty of Industrial Engineering, Robotics and Production Management, Technical University of Cluj-Napoca, Memorandumului 28, 400114 Cluj-Napoca, Romania; paul.bere@tcm.utcluj.ro

**Keywords:** Glass-Fiber-Reinforced Polymer (GFRP), drilling, cutting force, delamination, machine learning (ML)

## Abstract

The aim of the research presented in this paper was to simulate the relationship between selected technological drilling parameters (cutting speed, *v_c_*_,_ and feed per tooth, *f_z_*) and cutting forces and the delamination in machining of a new glass-fiber-reinforced polymer (GFRP) composite. Four different types of new materials were manufactured with the use of a specially designed pressing device and differed in the fiber type (plain and twill woven materials) and weight fraction (wf) ratio, but they had the same number of layers and the same stacking sequence. A vertical machining center Avia VMC800HS was used for drilling holes with a two-edge carbide diamond coated drill. Measurements of the cutting force *F_z_* in the drilling process conducted with variable technological parameters were carried out on a special test stand, 9257B, from Kistler. The new ink penetration method, involving covering the drilled hole surface with a colored liquid that spreads over the inner surface of the hole showing damage, was used to determine the delamination area. The cause-and-effect relationship between the drilling parameters was simulated with the use of five machine learning (ML) regression models (Linear Regression; Decision Tree Regressor; Decision Tree Regressor with Ada Boost; XGBRF Regressor; Gradient Boosting Regressor). Gradient Boosting Regressor results showed that the feed per tooth had the greatest impact on delamination—the higher the feed was, the greater the delamination became. Push-out delamination factors had higher values for materials that were made of plain woven fibers. The lowest amplitude of the cutting force component *F_z_* was obtained for the lowest tested feed per tooth of 0.04 mm for all tested materials, with the lowest values obtained for the materials with twill fibers.

## 1. Introduction

Composite materials, especially polymer composites, are widely used in almost every industry. Polymer composites are divided into thermosets and thermoplastics, and they can be reinforced by different types of fibers such as carbon fibers (CF), organic fibers, and glass fibers (GF) [1], etc. GFRPs are widely used in aerospace, aviation, automotive, wind turbine blades, and furniture industries [2,3] owing to their high mechanical properties, low density, high strength, and high corrosion resistance [4,5]. The properties of these materials depend on fiber properties, fiber type and amount, layer orientation, chemical stability, matrix strength, and interface bonding [6,7].

Polymer composites often require machining of composite parts at the final stage of production in order to meet the required shape and dimensional tolerances [8] and to create fitting and joining surfaces [9]. The machining of composite polymers often requires specialized tools and machining conditions, since they are classified as difficult-to-machine materials [10,11,12]. Conventional machining processes for GFRP parts are turning [13], milling [14], and drilling [15]. For the manufacture of various joints such as rivets, drilling is one of the methods to obtain holes characterized by the desired accuracy but requires appropriate tools and technological drilling conditions to prevent material failure. Damages caused by drilling are common and affect not only a component’s load carrying capacity but also its reliability. The most critical damage is delamination occurring in the drilling process because it causes heavy losses in different industries [16]. In the aircraft industry only, it is estimated that drilling-induced delamination accounts for about 60% of all part rejection at the final assembly stage for aircrafts [17].

The most common damage modes caused by machining GFRP composites are fiber pullouts, interlaminar delamination, fiber/matrix debonding, fuzzing, matrix melting, and softening [18]. Delamination occurring in the drilling process of GFRP composites reduces the structural integrity of the material and causes assembly tolerance error [19]. Delamination occurs when the tool leaves the material due to feed forces [20]. A combination of two mechanisms, mechanical and thermal damage, causes delamination in drilling FRP composite laminates. There are two types of delamination: peel-up delamination and push-out delamination. Peel-up delamination occurs at the entry plane when the fibers of the upper layers are not cut properly due to improper machining conditions (the first mechanism) and when the cutting edges of the tool contact the laminate. A peeling force is generated through the slope of a drill flute and peels away the top layers, causing peel-up delamination (the second mechanism) [21]. Push-out delamination occurs at the exit planes of the machined composite and is caused by both failure mechanisms because the drilled composite is loaded with axial and bending forces [22].

Many previous studies focused on factors influencing delamination. Generally, researchers investigate how machining parameters, tool materials, geometry, and tool types affect delamination. In relation to machining parameters, R. Bhat et al. [23] investigated the relationship between the influence of technological parameters (feed rate and speed) and GFRP samples thickness on the surface roughness and two types of delamination, peel-up and push-out delamination, in these materials. The first determining factor was the sample thickness and the other was the feed rate. Mohan et al. [24] investigated the influence of machining parameters such as speed and feed, as well as glass-fiber-reinforced orthophthalic polyester sample thickness (prepared with the contact molding process) and drill diameter on delamination. To determine the delamination on the upper and lower surfaces of each specimen, surfaces were scanned using a digital scanner and the delamination was determined by the measurements of the ratio of the delaminated area of the delamination zone to the ideal hole area. In the study, a coated carbide twist drill was used. They also found that the feed and material thickness had the greatest impact on both types of delamination. The most common tools used for drilling GFRP composites are polycrystalline diamond (PCD), coated or uncoated carbide, and coated or uncoated high-speed steel (HSS) drills. HSS drills are used because of economic reasons. Research has shown that PCD and carbide drills ensure a better hole surface quality than high-speed steel (HSS) tools [23]. A twill drill is frequently defined by a point angle. Many studies investigated the relation between the point angle and delamination. It was found that an increased point angle resulted in an increase in the extrusion area on the uncut layers of the laminate, resulting in a greater thrust force that caused push-out delamination. The use of low-angle drills in composite machining resulted in reduced push-out delamination [25,26,27].

Malik et al. [28] studied GFRP composite (fabricated using the vacuum infusion molding technique) drilling performance with different tools based on measurements of the thrust force, temperature, and delamination factor. The delamination factor was examined using a Mitutoyo 3D non-contact measuring system and defined as the ratio of the maximum diameter to the nominal diameter calculated using CAD software. According to the results, there is a relation between the thrust force and the machining parameters (feed and speed). The thrust force was lower at lower values of the speeds and feeds using the HSS drill bit. On the other hand, the lowest forces were obtained for solid carbide (SC) and solid carbide Balinit^®^ Helica-coated (SCBH) twist drills with a higher speed. Also Hy et al. [29] also found that in small-hole drilling of GFRP (manufactured by high-temperature molding), the lower thrust force values were obtained at a higher cutting speed and that peel-up and push-out delamination were lower too. A higher feed rate caused an increase in the thrust force and both types of delamination. The delamination factor defined as the ratio of the maximum diameter to the nominal diameter was assessed using 3D microscope Keyence (Osaka, Japan). A direct relationship was observed between the delamination factor and the feed rate and cutting speed. Devitte et al. [30] present the research results of the influence of cutting speed and cooled compressed air in the drilling of the GFRP/Ti-6Al-4V hybrid stack with an uncoated carbide drill, where GFRP was manufactured by vacuum bag molding. It was found that these two factors influenced the thrust force: an increase in the cutting speed reduced the thrust force by 28%. Conventional drilling can also include drilling pilot holes. The advantage of this technology is that the axial cutting force can be reduced, because the force is significantly lower during the second drilling, ensuring a higher hole quality [31]. Sun Z. et al. [32] analyzed the drilling performance of T800 CFRP composites using three different drills and found that for the dagger drill, the lowest thrust force and damages occurred. To further improve the drilling performance, ultrasonic vibration was successfully applied to dagger drills, and it resulted in the reduction in thrust force and surface roughness. According to the results, the combination of ultrasonic vibration and dagger drills is an effective method for drilling CFRP with high performance.

In addition to the influence of technological processing parameters, attention should also be paid to the influence of the manufacturing and assembly stages on residual stresses, because they reduce the strength of the structure and affect deformations. Vedernikov A.N. et al. [33] conducted an experiment involving measurements for 200 days after the manufacturing process of L-shaped pultruded FRP profiles produced with different pulling speeds in order to determine the deformation. On the basis of experiment results, it was found that curing at a slower rate results in lower shape distortions.

Due to increasing competition, the manufacturing industry is looking for new solutions that will reduce costs, save time, improve product quality, and reduce the amount of waste. An increasingly popular solution is the optimization of systems in manufacturing industries through the use of machine learning (ML). ML allows computers to learn from data and prior experiences, so that they can find patterns and predict future events without human interaction. ML is used in different engineering applications [34,35,36]. To enhance the sustainability of manufacturing processes and products, it is fundamental to predict nonlinear machining processes [37], and also in drilling process. A study [38] focused on the optimization of the delamination factor and torque in a drilling process for woven glass-fiber-reinforced epoxy composites. According to the results, the use of low feed rates and high spindle speeds led to a minimized delamination factor and maximized torque in the drilling process. A study [39] modeled the drilling parameters for GFRP using a machine learning technique with a back propagation (BP) multi-layer feed-forward network-based artificial neural network (ANN) model. According to the results, ANN models can be utilized in Industry 4.0 to predict the material removal rate during GFRP drilling. An analysis of the predictive models and the experimental results showed that the optimization process yielded good results, with an overall desirability factor of 90%.

Based on the literature analysis presented above, it can be concluded that there are many factors that influence the machinability of GFRP composites and their defects. The most important include technological parameters, the type of tool (material and its geometry), and the type of material and its dimensions (especially thickness). These factors are important to determine, especially when cutting new materials. This is why the purpose of this study was to analyze the relation between technological drilling parameters of drilling and cutting forces and the delamination types in the drilling of new GFRP composite types. The GFRP samples for this research were manufactured using an innovative method which was patented [40] and involves pressing the GFRP wet material between two rollers to eliminate the excess resin from the GFRP and is described in detail in [41]. The paper also describes a method for determining the delamination factor. This method is based on covering the surface of a drilled hole with ink which penetrates into mechanical processing-induced delaminated regions. This method enables the precise determination of the delamination factor. The results obtained with this method allow us to compare the influence of different technological parameters of drilling on delamination. Also, a relation between the cutting force and the technological parameters of drilling was established. The machinability of the fabricated GFRPs has not been investigated previously, but the results presented in this paper are the continuation of the research work presented in [41,42,43]. All those papers concern the study of the influence of drilling technological parameters on cutting forces for materials made on the basis of the same fibers, but with a different fiber content and made using different technologies (hand lay-up technology and application of a special pressing device). What is more, the cause-and-effect relationship between the drilling parameters was simulated with the use of machine learning. Understanding how these new materials can be machined is essential for potential industrial applications, because the selection of optimal technological parameters of drilling affects the cutting forces and the quality of drilled holes, thus reducing the number of rejected parts.

This research evaluated GFRPs because these materials are transparent, and the phenomenon of layer delamination can be observed much more easily. The results of this study can also be applied to other types of transparent composites. In this case, the phenomenon of delamination of the layers cannot be observed, with the material being opaque. The similarity between these materials can only be applied if the same matrix and the same type of architecture and the same type of wire of the reinforcing material are used. This can be determined by modern methods of non-destructive testing.

## 2. Materials and Methods

**Figure 1:**
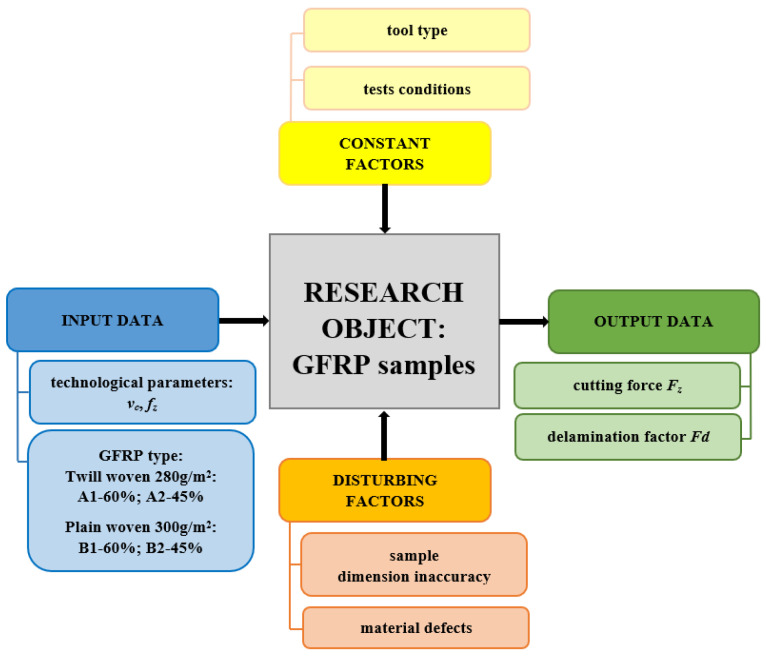
Research plan.

The aim of this study was to investigate the influence of selected technological drilling parameters (the cutting speed *v_c_* and the feed per tooth *f_z_*) and different types of GFRP materials (differing in fiber type and the wf ratios of reinforced material) on the cutting forces and delamination in this process, in order to obtain high-quality drilled holes. Optimal technological parameters for GFRP drilling, that meet quality requirements can be determined based on these results. Figure 1 illustrates the applied research plan.

### 2.1. Materials

This research was conducted using four different types of GFRP composites. The manufactured plates were labeled as A and B. The plates “A” were made of reinforced materials GF type E, Twill 2X2 woven by 280 g/m^2^, and EC9-3x68 Tex yarn type in warp. The “B” plates were made of reinforced materials GF type E, Plain woven by 300 g/m^2^, and the warp of the woven used was rowing’s by 300/300 tex. An epoxy resin of type EPIKOTE MGS LR135/LH 135 from Hexion Company, Esslingen am Neckar, Germany, was used for manufacturing both types of plates. The mixing ratio between resin and hardener was 100:35 parts by weight. The composite consisted of 4 fabric layers, where the layout was arranged in the configuration [0/90]_2_. Two different wf ratios of reinforced material were used to manufacture plates A and B by 60% and 45% (Table 1).

Wet technology was applied for all types of plates in order to impregnate the GF fabric using a new pressing device. A metal mold surface was covered with seven layers of chemical agents: 2 layers of Mold Sealer type S31 from Jost Chemicals Company, Kościan, Poland, and 5 layers of Frekote 770NC from Loctite Company, Düsseldorf, Germany. After applying each layer, the surface was allowed to dry for 10–15 min. After that, the GFRP was applied layer by layer to the surface of the mold, and after impregnation, it was covered with foil. The novelty of this technique lies in pressing the wet composite with a vacuum system without the use of a vacuum pump.

The technology used to obtain the GFRP plates presented in [40,41] consists of pressing the GFRP wet material between two rollers. The mold with the GFRP covered with foil is pressed by a roller to eliminate the excess resin from the GFRP. The rollers are rotated in opposite directions, and the plate goes through the gap between the rollers. The height of the gap can be adjusted depending on the thickness of the plate and composite. The rollers are driven by an electric motor with a reducer. In principle, the device operates like a calendaring installation. The mold is moved manually on a worktable equipped with rollers to the main rollers that move it as a result of rotation. Weight fraction (wf) ratios of the GFRP plates were controlled by the device which pressed the composite layers. By adjusting the space between the calendar cylinders, a greater or lesser pressing of the material was achieved. This determined the removal of a greater or lesser amount of resin.

The excess resin is removed from the mold edges after the resin passes through the cylinders. The novelty of this technology is the pressing of the composite material into the mold with an external force applied to the foil covering the GFRP material. This causes the excess resin and air bubbles to be moved towards the edge of the mold. The excess resin seals the edges, thus preventing the air from entering the composite material and changing its structure. A negative pressure is generated between the mold and the plastic foil, and there is no need to use a vacuum pump because the atmospheric pressure is used to press the composite material. Therefore, the material volume decreases, and the air cannot get into the material. The viscosity of the resin prevents the air from entering the composite in the edge area. After a short time, the resin turns into a gel, and the polymerization stage begins.

After that, the composites were left to cure at 22 °C for 24 h. Finally, a heat treatment was applied for 8 h at 80 °C.

### 2.2. Drilling GFRP

**Figure 2:**
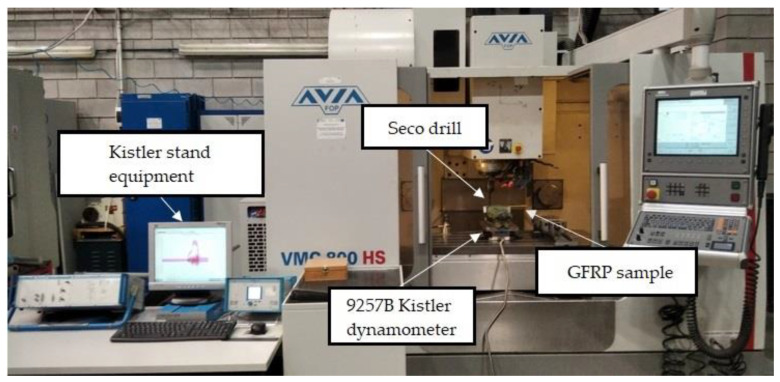
Test stand for measuring cutting force components.

A WaterJet Combo abrasive water jet cutter was used to cut the GFRP plates into samples for drilling. The samples had the dimensions 35 mm × 250 mm and the thickness of the sample depended on the type of composite. A total of 10 holes were drilled in each sample within the distance of 25 mm between the axis of the holes. A vertical machining center Avia VMC800HS (Avia, Warsaw, Poland) was used for drilling holes, and coolant was not applied. The drilling process was carried out using a 2-edge carbide diamond coated drill (SD205A-12.726-56-14R1-C2) of diameter 12.726 mm produced by Seco (Erkrath, Germany). During the tests, holes in the samples made of different types of GFRPs were drilled with variable drilling parameters: cutting speeds *v_c_* = 91, 182, 273, and 364 m/min and feeds per tooth *f_z_* = 0.04, 0.08, 0.12, and 0.16 mm/tooth. The drilling process was carried out without cooling. The technological parameters of drilling used in the tests were selected based on the results of preliminary tests. Five repetitions were performed for each parameter. In order to measure the cutting forces during the drilling process, a specially designed test stand was used. The test stand consists of 9257B dynamometer from Kistler (Winterthur, Switzerland), for measuring the components of cutting forces in three axes *F_x_*, *F_y_*, *F_z_*, a signal conditioning system, a DAQ module with an integrated A/D card, and dedicated software (Figure 2).

The cutting force amplitude value was taken as a difference between the maximum value and the minimum value of the force signal. The monitoring and control of cutting forces occurring during drilling is important for process improvement. It is also important from the point of view of critical parts safety because a thorough analysis of the drilling process based, among others, on cutting force measurements is an important factor influencing the maximum production reliability.

The standard deviation was calculated as

(1) $$s=\sqrt{\frac{\sum_{i=1}^{n}{\left(x_{i}-\bar{x}\right)}^{2}}{n-1}}$$

and presented in diagram form to show the scatter of the results. The amplitude value is calculated as the mean value in accordance with

(2) $$\bar{x}=\frac{\sum_{i=1}^{n}x_{i}}{n}.$$

where

$x_{i}$—each observation value;

$\bar{x}$—mean value;

n—number of values in the sample.

The next stage of the research involved delamination measurements.

### 2.3. Delamination Measurements Methods

One of the aims of this study was to determine two types of delamination: peel-up delamination, which occurs on the drill entry surface, and push-out delamination, which occurs on the surface when the drill exits the material. Given that the tested GFRP materials were produced with a new pressing device, it was important from the point of view of their industrial application to determine the impact of drilling on delamination.

**Figure 3:**
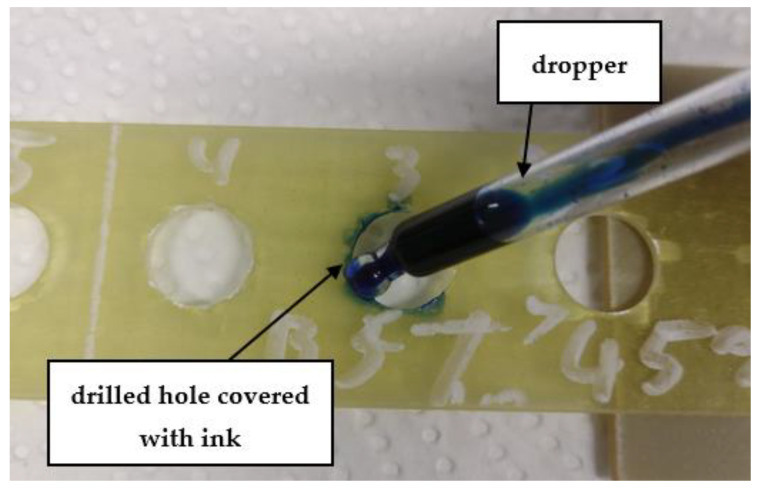
Method of covering the hole surface with ink.

Hole delamination was examined using a new ink method. The method consists of covering the surface of the hole with a colored liquid that spreads over the inner surface of the hole using a dropper. The method of covering the surface of the holes with ink is shown in Figure 3. The ink should be spread evenly over the hole surface. The liquid covers the surface, and damage is induced in the material inside the hole. In addition, the liquid penetrates into the delaminated area of the material, causing it to change its color. It is recommended to repeat the covering of the surface with ink, to cover all the delaminated areas. After applying each of two layers, wait 15 min for the ink to penetrate the delamination. After drying, clean the upper and lower surfaces of the sample from excess ink by wiping it with a cloth soaked in ethyl alcohol.

The method allows easy identification of the delamination area. This is especially important for GFRP materials where delamination is not always easy to detect. The phenomenon of delamination is, however, very easy to detect in fabricated GFRP parts, which can be attributed to the transparency of the plates. The conclusions of this study can be extended to other types of composites such as CFRP or AFRP. This is if the type of fabrics or fabrics used is respected. In the case of another type of composite, the delamination cannot be observed.

**Figure 4:**
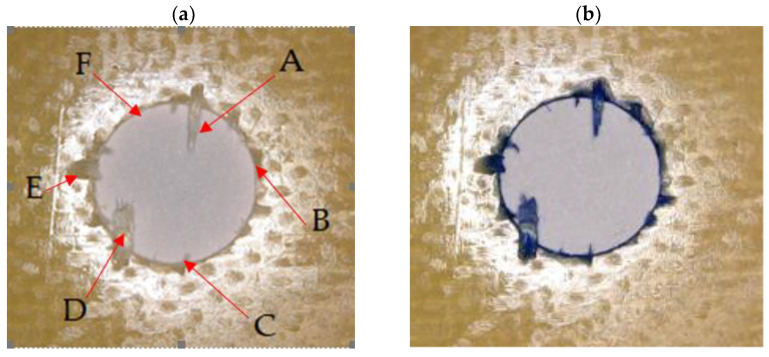
Images of peel-up delamination of holes drilled in material A1. (**a**) Clear delamination image, (**b**) liquid-colored delamination, with marked damages: A—spalling and pulled out fibers; B—spalling; C—pulled out fibers; D—spalling and matrix debonding; E—spalling and uncut fibers; F—uncut fibers.

For research purposes, a mixture of navy blue ink and ethyl alcohol was used (respectively, in the mixing ratio of 50:50). An example of a hole (A1 material) with a push-out delamination area without ink and ink-colored is shown in Figure 4. The figure presents the types of damage occurring around the drilled hole. A Keyence VHX-5000 optical microscope at 10× magnification was used to capture images.

**Figure 5:**
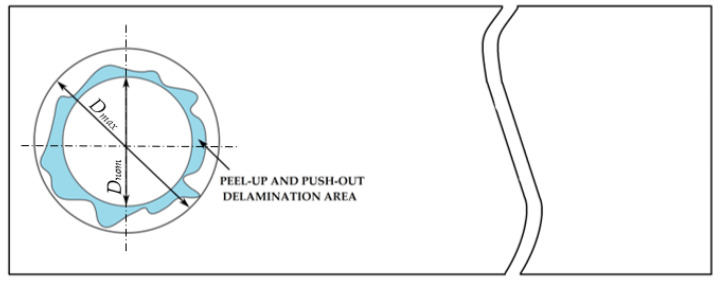
Determination of the delamination factor.

The delamination factor is calculated as [41,44]

(3) $$F_{d}=\frac{D_{max}}{D_{nom}},\;[-]$$

where $F_{d}$—delamination factor; $D_{max}$—maximum delaminated diameter drawn from the centerline of the hole; and $D_{nom}$—nominal diameter (according to Figure 5).

The nominal and maximum delaminated diameters were measured with the Keyence VHX-5000 optical microscope software at 10× magnification.

### 2.4. Machine Learning

The main goal of creating the model is to estimate the *F_z_* parameter based on the type of material, the *v_c_* parameter, and the *f_z_* parameter. Only the component of the cutting force *F_z_* was an object of prediction models, because it plays the largest role in the drilling process and assumes the largest value. For this purpose, a data set was created consisting of previously indicated parameters. The dataset was divided in the proportions of 7:2:1 into a training, validation, and test set. In order to create a regression model for obtained data, the following methods were employed:

- Linear Regression;
- Decision Tree Regressor;
- Decision Tree Regressor with Ada Boost (Drucker, 1997);
- XGBRF Regressor;
- Gradient Boosting Regressor.

For the Decision Tree Regressor model, the maximum depth of the tree was defined as the point at which all leaves are pure or at which fewer than two samples are present in each leaf. Squared error was chosen as the function to measure the quality of split in this scenario. When utilizing the Ada Boost algorithm, the decision tree’s parameters were the same with the exception of the maximum depth, which was taken to be 3. Additionally, a parameter that indicated the number of boosting iterations—which was taken to be 100—was used. In the case of using the XGBRF Regressor algorithm, the number of iterations was also 100, the learning rate parameter was 1, and the division into subsamples was 0.8. For the Gradient Boosting Regressor model, the squared error was taken as the loss function, with a learning rate of 0.1, the target number of boosting stages to perform was 100, Friedman MSE was used as the function measuring the quality of the split, and the maximum depth of individual estimators was defined as 3.

To compare the performance of the models, the following metrics were used: coefficient of determination—R^2^; mean absolute error (MAE); and Root-Mean-Square Error (RMSE). Equations (4)–(6) represent the metrics previously described.

(4) $$R^{2}=\frac{{\sum_{i=1}^{i}\left(\hat{y_{i}}-y_{i}\right)}^{2}}{{\sum_{i=1}^{i}\left(y_{i}-\hat{y_{N}}\right)}^{2}}$$

(5) $$MSE=\frac{1}{i}\sum_{i=1}^{i}{\left(\hat{y_{i}}-y_{i}\right)}^{2}$$

(6) $$RMSE=\sqrt{\frac{1}{i}\sum_{i=1}^{i}{\left(\hat{y_{i}}-y_{i}\right)}^{2}}$$

where

$y_{i}$—i-th ground truth value;

$\hat{y_{i}}$—i-th predicted value;

i—total number of samples.

## 3. Results and Discussion

The results of the cutting force and delamination factor analysis obtained in drilling for different types of GFRP samples are presented below.

### 3.1. Cutting Forces

Due to the fact that the share of the cutting force component *F_z_* is the largest in the drilling process compared to other components (*F_x_* and *F_y_*), this section discusses the results of this component.

**Figure 6:**
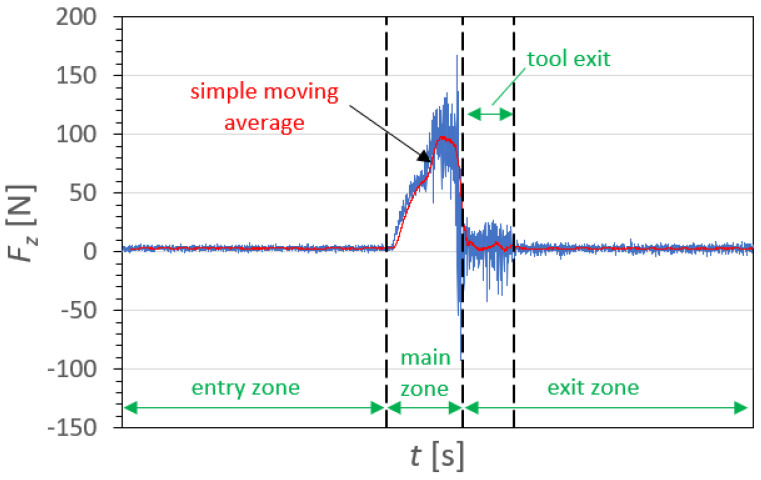
Example of a time course of the cutting force component *F_z_* for material A1, for a drilling process conducted with the following technological parameters: cutting speed *v_c_* = 273 m/min, feed per tooth *f_z_* = 0.12 mm/tooth.

An example of the characteristics of the course of changes in the cutting force component *F_z_* as a function of the time t for sample A1, in a drilling process conducted with *v_c_* = 273 m/min and *f_z_* = 0.12 mm/tooth, is shown in Figure 6. It can be observed that the cutting force component *F_z_* (also known as a feed force or thrust force) takes both positive and negative values. *F_z_* plays a major role in the drilling process. Other components of the cutting force *F_x_* and *F_y_* take lower values, which was confirmed by the research presented in [43], which concerned GFRP composites manufactured based on the same fibers as in this paper with a range of different technological parameters. Three zones can be identified in the force course: the entry zone, main drilling zone, and exit zone. At the beginning of the process, in the entry zone, the cutting force oscillates around 0 N, taking the lowest values below 10 N. In the main drilling zone, the drill enters the workpiece, causing an almost linear increase in the cutting force component *F_z_*, and its value stabilizes over time due to full engagement of the cutting edges [15]. This is followed by a decrease in the cutting force component *F_z_* due to fewer layers to be cut. In the exit zone, the tool exits the workpiece, which causes a decrease in the cutting force component *F_z_*. Periodic peaks can be observed in the force curve when the composite fibers are cut. Figure 6 also shows the Simple Moving Average (SMA) to smooth the *F_z_* course pattern.

**Figure 7:**
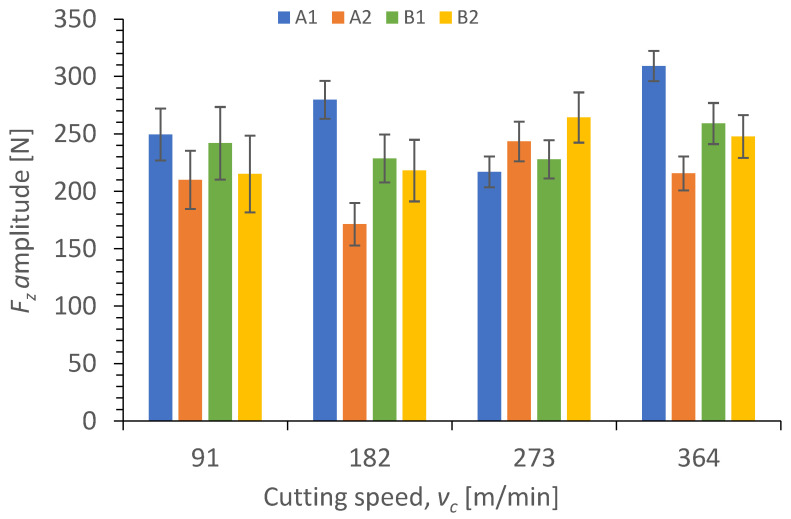
Relationship between the amplitude of cutting force component *F_z_* and the cutting speed *v_c_* in a drilling process conducted with a with constant feed per tooth of *f_z_* = 0.12 mm/tooth for different GFRP materials.

**Figure 8:**
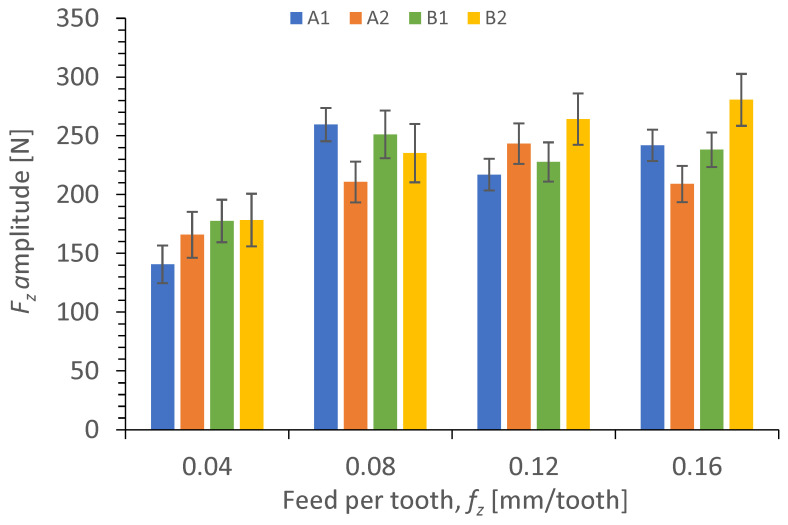
Relationship between the cutting force component *F_z_* amplitude and the feed per tooth *f_z_* in a drilling process conducted with a cutting speed of *v_c_* = 273 m/min, for different GFRP materials.

Results illustrating a relationship between the amplitude value of cutting force component *F_z_* and the technological cutting parameters in the drilling process of the four types of GFRP composites are presented in Figure 7 and Figure 8.

The relationship between different cutting speeds *v_c_* and the cutting force component *F_z_* amplitude for four types of GFRP materials drilled with a constant feed per tooth of *f_z_* = 0.12 mm/tooth is shown in Figure 7. The maximum value of the cutting force component *F_z_* amplitude was obtained at the highest value of the cutting speed *v_c_* for material A1 and was equal to 309 N. The minimum value of 171 N was obtained for the A2 material at the cutting speed *v_c_* equal to 182 m/min. A comparison of the samples made of the same material but differing with the wf ratios of reinforced material reveals the following relationships. For almost every cutting speed value (except *v_c_* = 273 m/min), there is a noticeable decrease in the cutting forces component *F_z_* for the A2 material. For the cutting speed *v_c_* = 91 m/min, the difference in the *F_z_* values of the two materials is 16%, for *v_c_* = 182 m/min it is 38%, and for *v_c_* = 364 m/min it is 30%. This is due to the fact that for the A1 material containing a higher value of the weight fraction ratio of reinforced material, the forces needed to drill a hole and overcome the resistance of the fibers are higher than for the composite with a lower wf. An increase in the fiber volume fraction increases the thrust forces [45]. Considering the amplitudes of the cutting force component *F_z_* for materials B1 and B2, taking into account the standard deviation, we cannot conclude that there are significant differences between the values obtained for these two materials characterized by different wf ratios of reinforced material.

The relationship between the cutting force component *F_z_* amplitude and different feeds per tooth *f_z_* in the drilling process for GFRP composites is presented in Figure 8.

Figure 8 shows the effect of feed per tooth *f_z_* on the cutting force component *F_z_* amplitude for different types of GFRPs. For each tested material, the amplitude of the cutting force component *F_z_* increases with increasing the feed per tooth *f_z_*. Other studies [41] also found that the feed rate had the greatest impact on the cutting force in drilling. The lowest amplitude values of the cutting force component *F_z_* were achieved for the lowest tested feed per tooth value of 0.04 mm/tooth for all tested materials (A1 = 140 N, A2 = 165 N, B1 = 177 N, B2 = 178 N), similar to in study [43]. A comparison of the lowest (*f_z_* = 0.04 mm/tooth) and the highest feed per tooth (*f_z_* = 0.16 mm/tooth) demonstrates that the highest increase in the cutting force component *F_z_* amplitude (about 71%) was obtained for material A1, then for B2 (57%), and finally for B1 (34%), while it was the smallest for A2 (the amplitude value increased by about 26%). This means that the feed per tooth has the smallest impact on the cutting force component *F_z_* for the A2 material made of twill fibers and with a lower wf ratio of reinforced material. Increasing the feed rate increases the material removal rate (MRR) for all tested materials. The higher MRR means a higher volume to cut, which leads to higher friction because of the higher contact area between the cutting tool face and the workpiece causing the higher force.

### 3.2. Delamination of GFRP Materials

**Figure 9:**
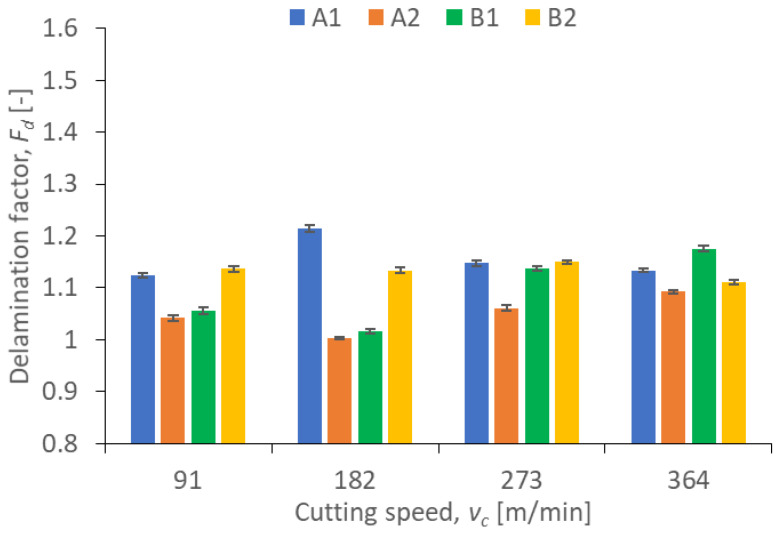
Relationship between peel-up delamination factor *F_d_* and cutting speed *v_c_* in a drilling process conducted with a constant feed per tooth of *f_z_* = 0.12 mm/tooth, for different GFRP materials.

**Figure 10:**
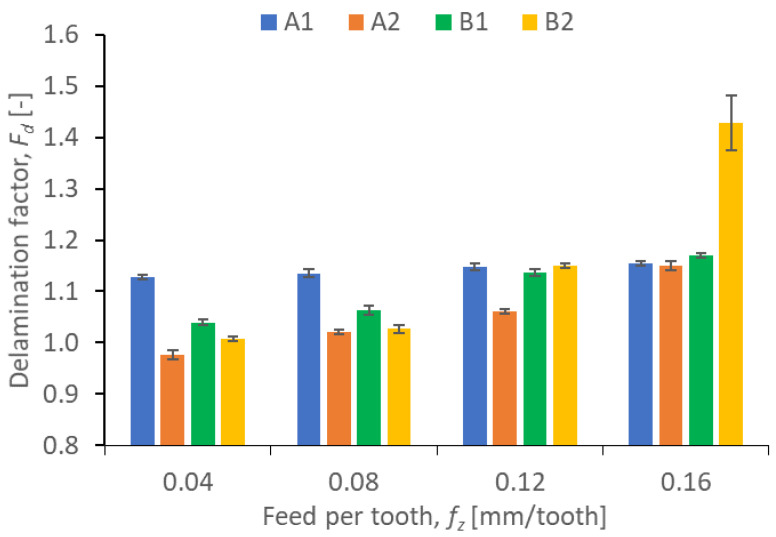
Relationship between peel-up delamination factor *F_d_* and feed per tooth *f_z_* in a drilling process conducted with a constant cutting speed of *v_c_* = 273 m/min, for different GFRP materials.

**Figure 11:**
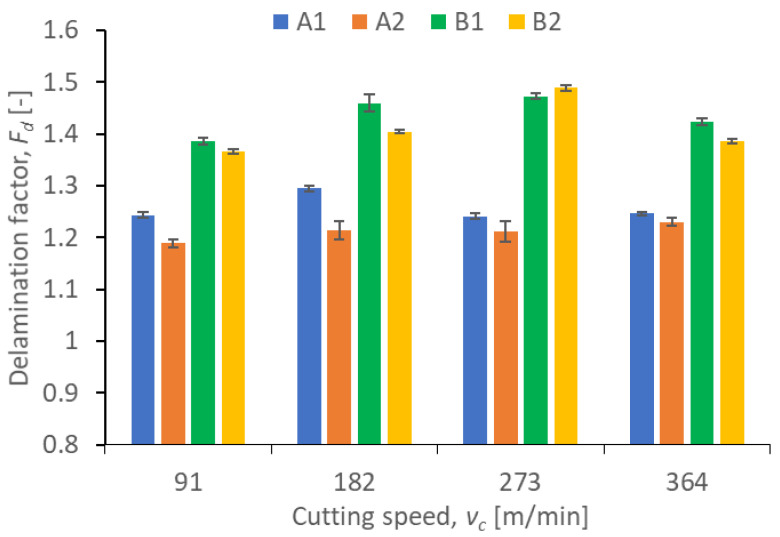
Relationship between the push-out delamination factor *F_d_* and the cutting speed *v_c_* in a drilling process conducted with a constant feed per tooth of *f_z_* = 0.12 mm/tooth, for different GFRP materials.

**Figure 12:**
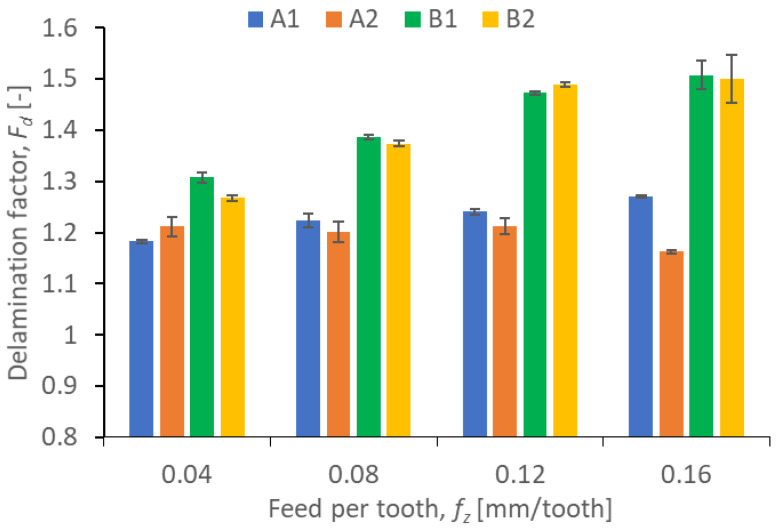
Relationship between push-out delamination factor *F_d_* and feed per tooth *f_z_* in a drilling process conducted with a constant cutting speed of *v_c_* = 273 m/min, for different GFRP materials.

Results illustrating the relationship between peel-up and push out delamination factors and certain technological cutting parameters (*v_c_* and *f_z_*) in the drilling process for four types of GFRP composites are presented in Figure 9, Figure 10, Figure 11 and Figure 12. The delamination factor was calculated in accordance with Equation (1).

The relationship between the peel-up delamination factor and the cutting speed is presented in Figure 9. It can be observed that the lowest values of the peel-up delamination factor were obtained for A2 (*F_d_* = 1.003) and B1 (*F_d_* = 1.017) materials with *v_c_* = 182 m/min. For these materials, an increase in the cutting speed (in the range *v_c_* = 182 ÷ 364) caused an increase in the delamination factor value by 8.87% for A2 and by 15.43% for B1. This is an almost linear increase, and similar to the findings reported in [42], where an increase in the peel-up delamination factor was observed with an increase in the cutting speed. The highest value of the peel-up delamination factor amounting to 1.214 was observed for the A1 material with *v_c_* = 182 m/min. For other cutting speeds for this material, the delamination factor values were comparable, ranging from 1.12 to 1.14. For the B2 samples, the peel-up delamination factor values were also comparable, ranging from 1.11 to 1.15. In case of the A1 material, we can clearly notice the influence of the wf ratio on the delamination factor. A1 characterized by a higher wf ratio of reinforced material causes the higher values of the peel-up delamination factor in the range of all cutting speeds.

Figure 10 shows the peel-up delamination factor values in a drilling process conducted with different feeds per tooth *f_z_* and a constant cutting speed *v_c_* for different types of GFRP. The delamination factor values are the lowest for all the materials drilled with the lowest feed values (A1 = 1.13, A2 = 0.98, B1 = 1.04, B2 = 1.01). According to the diagram, the feed per tooth plays a significant role in the delamination factor for all GFRP samples, since the delamination factor increases with increasing the feed per tooth, which is consistent with the results reported in [43]. A comparison between the lowest and the highest feed per tooth shows that the smallest increase in the delamination factor amounting to about 1.76% is obtained for A1, then for B1 (12.49%), and for A2 (17.34%), while the highest increase is obtained for the B2 material (41.58%). The materials with the lowest increase in the delamination factor with increasing the feed per tooth are characterized by the same wf ratios of reinforced material amounting to 60%. In Figure 10, we can observe that the drilling-induced delamination rises by increasing the feed rate for most of materials. This mechanism can be related to the rise of the axial thrust force due to expanding the cross-sectional area, which also increased when the feed was increased. The higher feed rate creates more friction, which in turn, increases the delamination factor [46].

The data in Figure 11 show that the largest increase in the push-out delamination factor versus the cutting speed can be observed for materials B1 and B2. For B1 and B2, in the cutting speed *v_c_* range of 91 ± 273 m/min, the push-out delamination factor increases, reaching the maximum at the cutting speed *v_c_* of 273 m/min first, and then, it begins to decrease. For the B2 material, this is an increase of 8.82% and for the B1 material of 6.52% (comparing the cutting speeds *v_c_* of 91 and 273 m/min). These materials are made of the same plain woven fiber but differ in the weight fraction ratio of reinforced material. We can therefore conclude that this is one of the indicators affecting delamination. For A1 and A2 materials, the increase in the push-out delamination factor is smaller and amounts to 4.03 and 4.23%, respectively (comparing the minimum and the maximum push-out delamination values at different cutting speeds). Therefore, it can be concluded that the A1 and A2 materials are characterized by lower delamination when the drilling process is conducted using a variable cutting speed.

A comparison of the results in Figure 9 and Figure 11 for peel-up and push-out delamination reveals that the peel-up delamination factor is lower for each tested material compared to the push-out delamination factor for the same material, regardless of the cutting speed. From the point of view of the delamination factor, the type of fiber also seems to be important. The materials marked as B, containing Plain woven by 300 g/m^2^ fibers, were characterized by the highest push-out delamination index, regardless of the adopted cutting parameters. This means that these materials are more susceptible to delamination in the tool exit zone. This is caused by the tool pushing out the last layers of the composite.

Based on Figure 12 showing the relationship between the delamination factor and feed, it can be observed that the feed is an important indicator affecting the push-out delamination. For three out of four analyzed materials, the delamination factor increases with increasing the feed rate, which can be related to the rise in the axial thrust force. The increase is, respectively, 7.63% for material A1, 15.27% for material B1, and 18.11% for material B2. Only in the case of material B1 did the increase in feed cause a decrease in the delamination factor by 4.13%, comparing the lowest and the highest feed value. The push-out delamination factors were higher for materials from group B, regardless of the values of technological cutting parameters (feed per tooth and cutting speed).

Since the delamination factor predominantly depends on the feed per tooth, in Table 2 and Table 3 the images of the drilled holes with delamination area are shown, for the samples with and without the application of ink in the drilled hole surfaces.

Based on the above images (Table 2) captured with the Keyence microscope without ink and with the use of the new delamination detection method involving the introduction of ink on the drilled hole surface, it is possible to see many positive aspects of this method. Firstly, this method allows for easy estimation of the delamination factor, because the ink easily penetrates into the delaminated areas and marks them with color. This facilitates the determination of the delamination area necessary to determine the delamination factor. Secondly, this method also allows you to quickly identify other damage modes, e.g., torn fibers, because they are colored and instantly visible under the microscope. This method for delamination determination is important, especially in the case of materials based on glass fibers, where delamination is difficult to detect on the surface of the sample due to its color. The images also confirm that the delamination in drilled GFRP composites increases with the increasing feed per tooth rate.

Based on the images shown in Table 3 for the push-out delamination in holes drilled in four different types of GFRP samples at a constant cutting speed and different feeds per tooth, we can observe that the application of the ink method works differently when it comes to determining push-out delamination. The ink introduced on the surface of the hole penetrates the delaminated region, and it is thus easy to identify by color marking. This can be observed, for example, by comparing the images for samples B1 at the maximum feed per tooth value *f_z_* = 0.16 mm/tooth (d), where the introduced ink precisely colors the delamination range. For the holes photographed without ink, it is difficult to determine whether the different color of the sample is due to delamination or microscope illumination of the sample.

### 3.3. Machine Learning Models

**Figure 13:**
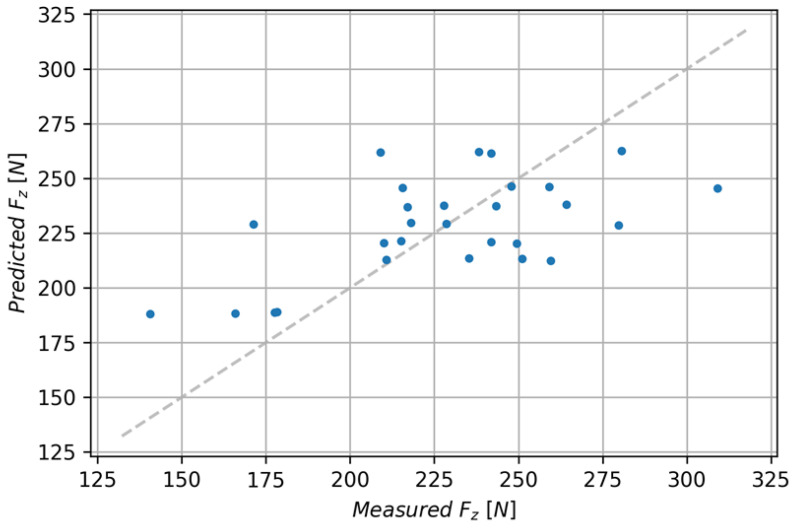
Comparison of predicted and measured values for the linear regression model.

To compare the performance of the models, the following metrics were used: the coefficient of determination—R^2^; mean absolute error (MAE); and Root-Mean-Square Error (RMSE). The linear regression model had the lowest coefficient of determination (0.352), it also achieved the highest average absolute error of 24.05 and the highest Root-Mean-Square Error of 29.92. Figure 13 shows the comparison of predicted and measured values for the discussed model.

**Figure 14:**
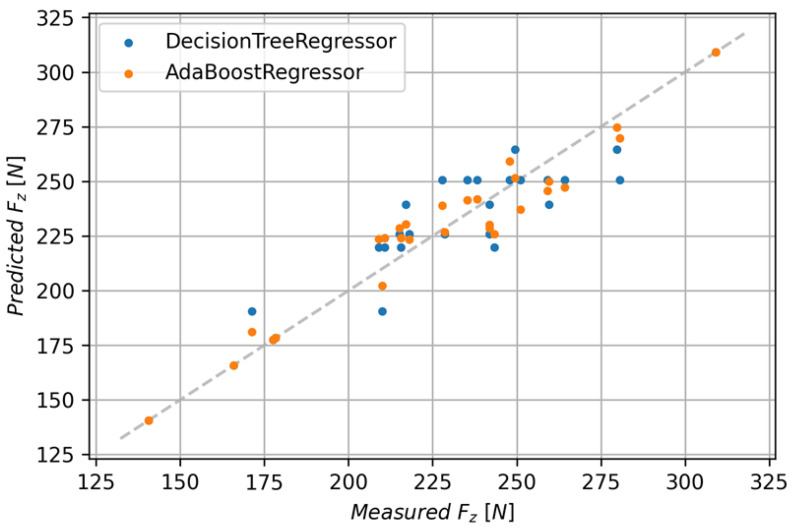
Comparison of predicted and measured values for the Decision Tree Regressor and Decision Tree Regressor with Ada Boost.

The Decision Tree Regressor model achieved an R^2^ score of 0.860 with an RMSE of 13.89 and MAE of 10.87. In order to improve the performance of the model, the Ada Boost algorithm was used. This allowed for an improvement of the R^2^ score to 0.927 with a mean absolute error of 8.37 and RMSE of 10.07. Figure 14 shows the comparison of predicted and measured values for both discussed models.

**Figure 15:**
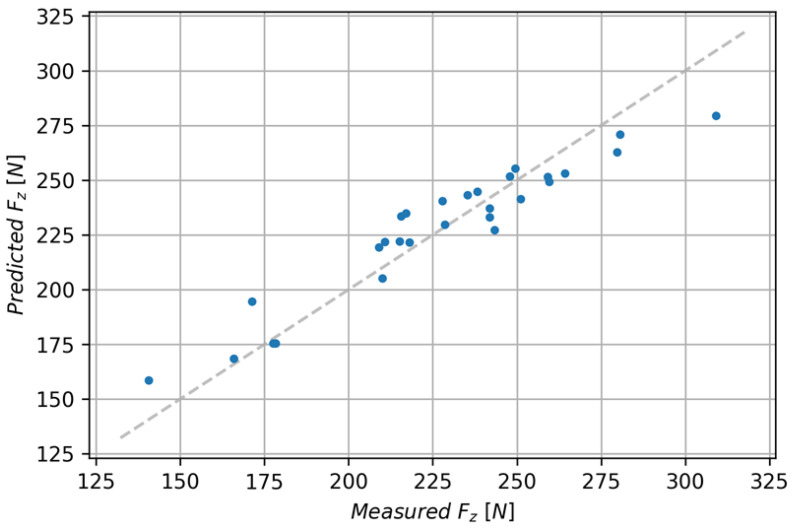
Comparison of predicted and measured values for the XGBRF Regressor.

The XGBRF Regressor model achieved lower metrics than the previous models. This model has an R^2^ of 0.893 with an MAE of 10.16 and a Root-Mean-Square Error of 12.18. Figure 15 shows a comparison of predicted and measured values for both discussed models.

**Figure 16:**
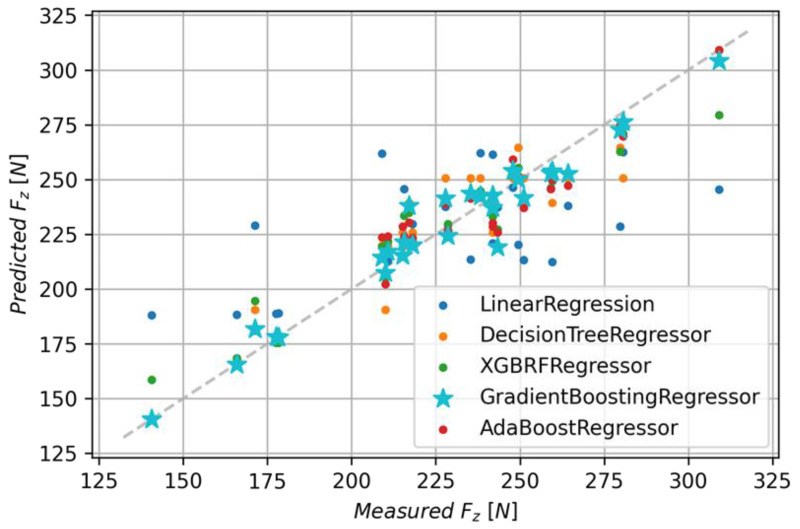
Comparison of predicted and measured values for all models.

The Gradient Boosting Regressor model has the best metrics values. It achieved the coefficient of determination of 0.948 with an MAE of 6.13 and RMSE of 8.46. A comparison of this model and the other ones is given in Figure 16, and Table 4 lists a summary of metrics for each model.

**Figure 17:**
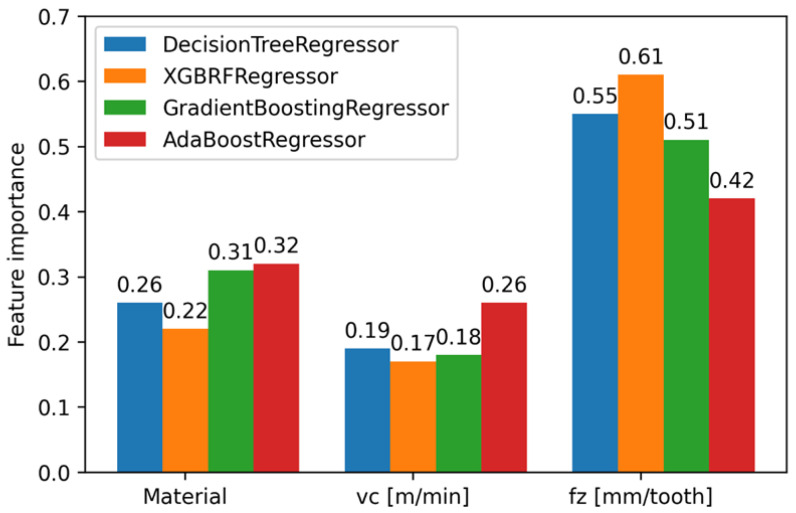
Feature importance for selected models.

For the models presented in Table 4, whose R^2^ score was greater than 0.5, an analysis of the importance of features was performed. The Ada Boost Regressor model among the tested models shows the least importance for the *f_z_* parameter, which is 0.42, and the XGBRF Regressor model assigned the highest importance of 0.61 for this parameter. The Gradient Boosting Regressor model assigned an importance value of 0.51 to the *f_z_* parameter, 0.18 to the *v_c_* parameter, and an importance value of 0.31 for the material type. The importance of features for the indicated models is shown in Figure 17.

**Figure 18:**
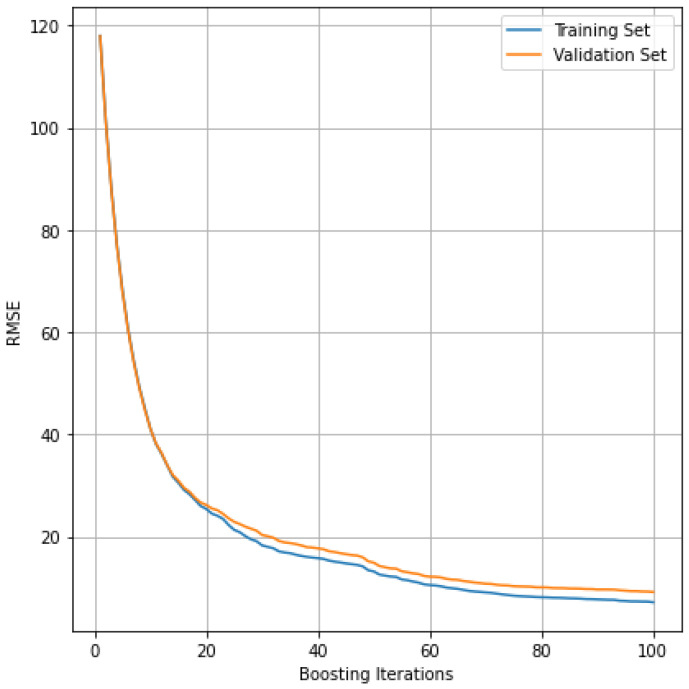
The RMSE value per iteration.

The target model, Gradient Boosting Regressor, was selected due to the largest R^2^ score metric and the smallest MAE and RMSE metrics. In order to check that the model does not exhibit overfitting, the RMSE value curve for a given iteration is presented in Figure 18.

**Figure 19:**
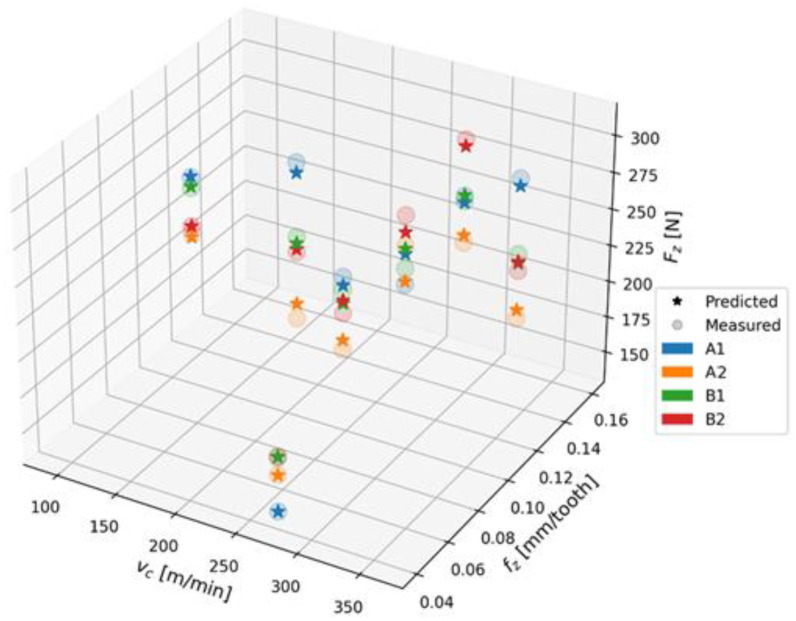
Chart comparing measured and predicted values for Gradient Boosting Regressor model.

As can be seen from the chart presented above, the model does not show any tendency to overfitting. The feature value for both the training and test sets is minimized. In order to present the differences between the measurements and the values predicted by the Gradient Boosting Regressor model for the test set, a 3D chart was created (Figure 19). The Gradient Boosting Regressor model for the test set achieved an R^2^ score of 0.931, with an MSE of 6.10 and RMSE of 8.43.

## 4. Conclusions

The novelty of this study consists of a method for determining delamination by introducing ink on the surface of the drilled hole in four types of GFRP samples and the study of the machinability of these materials. Since our study is the first to use the ink method for delamination determination and to test the machinability of these new GFRP materials, it fills the knowledge gap in this field. In addition, the optimal parameters of the drilling process ensuring reduced delamination were determined.

The study investigated GFRP materials that were fabricated with different wf ratios using an epoxy matrix made by a special technique and specified machining parameters. These aspects have not yet been investigated from the point of view of delamination of the drilling process.

The results of the study investigating cutting forces and delamination in GFRP drilling lead to the following conclusions:

- The new method for assessing delamination by applying ink on the surface of the drilled hole to penetrate the delamination area can be used for both peel-up and push-out delamination assessment. This method also allows for the easy identification of other damage modes, e.g., fiber pullouts, which may go unnoticed due to the color of GFRP composites;
- The method of assessing delamination by applying ink on the composite surface can potentially be used to assess elements of aircraft structures made of GFRP that have undergone, for example, mechanical damage in order to assess delamination and further qualify these parts for repair or replacement;
- The factor having the greatest impact on delamination is the feed rate; the higher the feed rate, the greater the delamination becomes. This mechanism can be related to the rise in the axial thrust force due to the expanding cross-sectional area, which also increased when the feed was increased;
- Push-out delamination has a greater range than peel-up delamination, regardless of the tested material;
- The push-out delamination factors were higher for materials from group B, regardless of the technological cutting parameters (feed per tooth and cutting speed). A comparison of the lowest and the highest feed per tooth values for materials A1 and B1 with different fiber types but the same weight fraction ratios of reinforced material demonstrated that the delamination factor increased by 18.11% for B1 and by 7.63% for A1. It has been found that delamination depends on the type of fabric used. The thicker the type of fabric and the thicker the threads are (threads made up of a larger number of monofilaments), the more pronounced the delamination becomes;
- The cutting force *F_z_* in the drilling process primarily depends on the feed rate, rather than on the cutting speed. The amplitude of the cutting force component *F_z_* increases with the increasing feed per tooth *f_z_*;
- The lowest amplitude values of the cutting force component *F_z_* were achieved for the lowest tested feed per tooth value of 0.04 mm/tooth for all tested materials (A1 = 140 N, A2 = 165 N, B1 = 177 N, B2 = 178 N). The cutting force was the highest for type B materials that also showed the highest push-out delamination factor;
- A comparison of the lowest and the highest values of feed per tooth demonstrated that the largest increase in the amplitude of the cutting force component *F_z_* of about 71% was obtained for material A1, it was 57% for B2 and 34% for B1, while the lowest *F_z_* increase of 26% was obtained for A2. This means that the feed per tooth has the lowest impact on the cutting force component *F_z_* in the case of the A2 material made of twill fibers and characterized by a lower wf ratio of reinforced material;
- Material A2 made of twill woven fibers, containing 45% of reinforced material, is characterized by the lowest delamination factor, regardless of the type of delamination and technological parameters applied in tests. This also indicates that delamination depends on the wf ratio of reinforced material on delamination;
- The Gradient Boosting Regressor model has the best metric values from all analyzed models. It achieved the coefficient of determination of 0.948 with an MAE of 6.13 and RMSE of 8.46, which implies that machine learning techniques are a suitable tool for modeling the cutting force component *F_z_* as a function of technological parameters. One of the potential applications of the Gradient Boosting Regression model in the industry is to predict the value of the *F_z_* parameter before starting the process, which will allow for optimizing the selection of cutting conditions from the point of view of its energy consumption and minimizing delamination processes. Knowledge of *F_z_* allows for predicting the occurrence of this phenomenon and controlling the process in such a way as to obtain the smallest possible defects in the holes made.

Based on the obtained results, it can generally be concluded that lower cutting forces occur for type A materials made of twill type fibers, at lower feed per tooth values, and that these materials are also characterized by lower push-out delamination. The delamination detection method by introducing ink into the surface of the drilled hole is an effective method for assessing the damage to GFRP composites.

Future studies can investigate the problem of delamination with respect to thinner or thicker layers of reinforcing material (with higher mass and thicker threads), the use of drill types with special geometry, different material thicknesses or matrix types, as well as process parameters of different manufactures. The conclusions of this study can be a simplifying hypothesis for other types of FRP having the same architectural arrangement of the layers and the same type of fabric or matrix. The authors also plan to conduct research on a new method of determining delamination using ink, by comparing its effectiveness with conventional methods of assessing delamination, so that it can be used in aviation to assess delamination of composite parts after mechanical damage.

## Figures and Tables

**Table 1:** Four types of GFRP used in the study.

Notation	A1	A2	B1	B2
wf [%]	60	45	60	45
Thickness [mm]	1.2	1.7	1.3	1.8
Number of layers	4			

**Table 2:** Microscopic images of peel-up delamination in holes with and without ink for all types of GFRP samples, drilled with a constant cutting speed of *v_c_* = 273 m/min and different feeds per tooth *f_z_*: (**a**) *f_z_* = 0.04 mm/tooth, (**b**) *f_z_* = 0.08 mm/tooth, (**c**) *f_z_* = 0.12 mm/tooth, (**d**) *f_z_* = 0.16 mm/tooth.

	(a)	(b)	(c)	(d)
A1	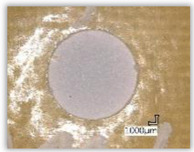	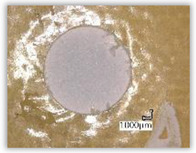	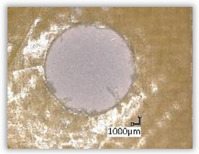	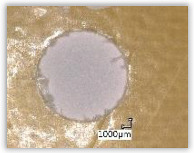
A1 with ink	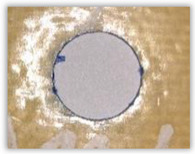	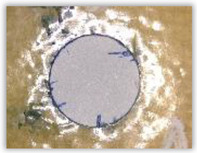	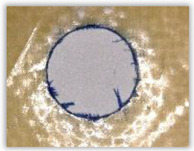	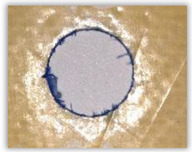
A2	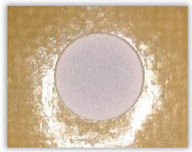	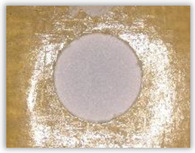	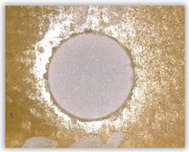	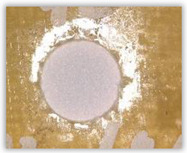
A2 with ink	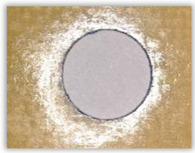	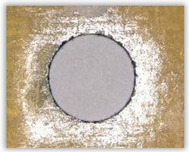	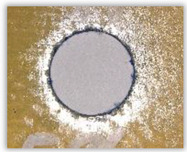	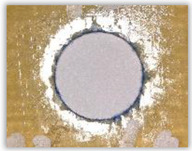
B1	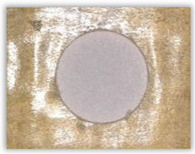	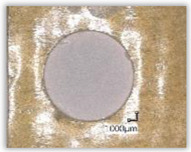	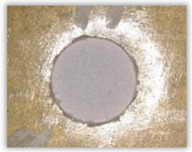	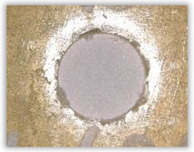
B1 with ink	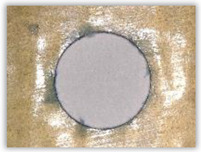	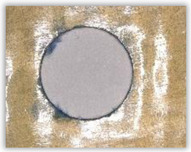	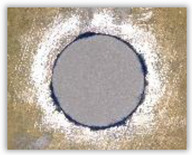	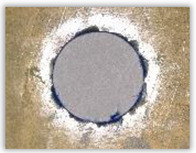
B2	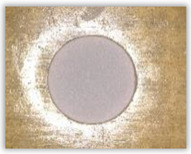	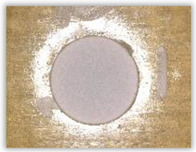	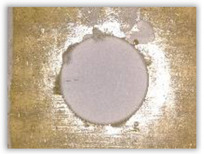	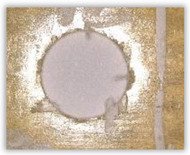
B2 with ink	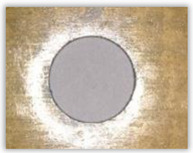	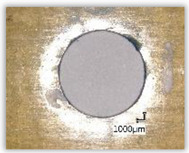	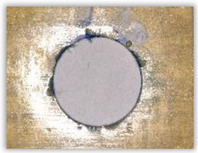	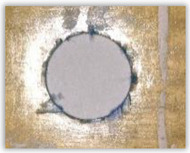

**Table 3:** Comparative photos of push-out delamination taken under a microscope for holes covered with ink and without for each type of GFRP sample drilled with constant cutting speed of *v_c_* = 273 m/min and different feed per tooth *f_z_* values: (**a**) *f_z_* = 0.04 mm/tooth, (**b**) *f_z_* = 0.08 mm/tooth, (**c**) *f_z_* = 0.12 mm/tooth, (**d**) *f_z_* = 0.16 mm/tooth.

	(a)	(b)	(c)	(d)
A1	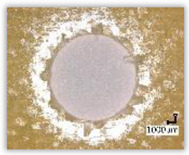	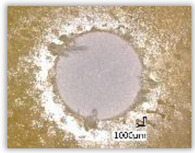	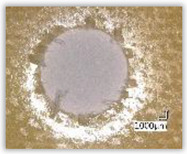	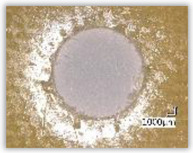
A1 with ink	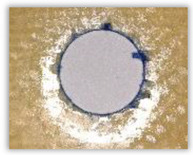	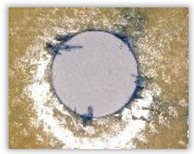	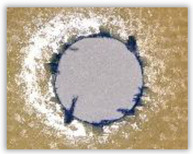	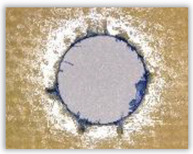
A2	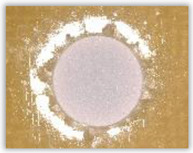	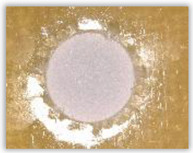	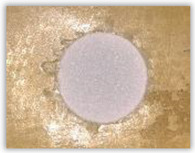	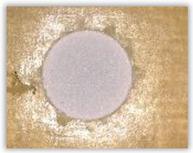
A2 with ink	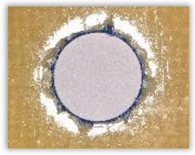	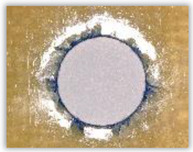	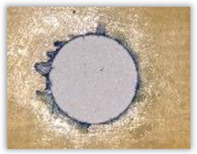	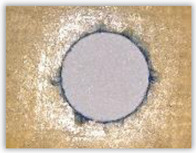
B1	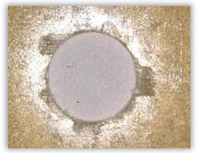	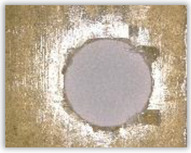	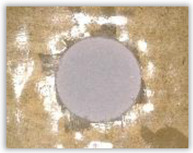	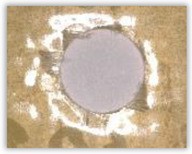
B1 with ink	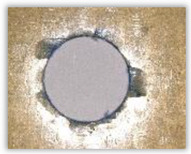	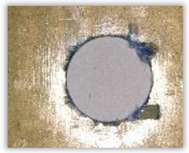	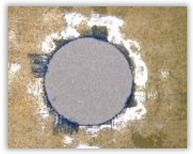	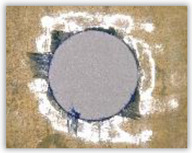
B2	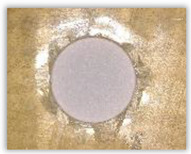	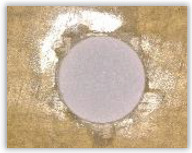	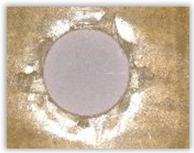	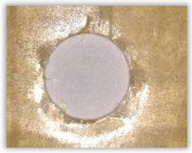
B2 with ink	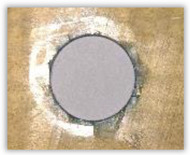	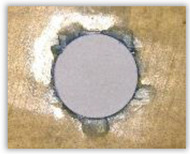	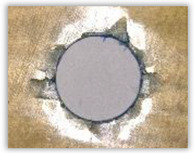	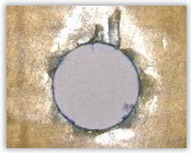

**Table 4:** Model metrics.

Model	R^2^	MAE	RMSE
Linear Regression	0.352	24.05	29.92
Decision Tree Regressor	0.860	10.87	13.89
Ada Boost Regressor	0.927	8.37	10.07
XGBRF Regressor	0.893	10.16	12.18
Gradient Boosting Regressor	0.948	6.13	8.46

## Data Availability

Data are contained within the article.
